# Successful use of clozapine in a patient with schizophrenia comorbid with 22q11.2 deletion syndrome and multiple periventricular nodular heterotopia: A case report

**DOI:** 10.1002/pcn5.70195

**Published:** 2025-08-25

**Authors:** Kiwamu Hoshi, Koichi Matsuyama, Yasunori Oda, Shintaro Shibata, Teruomi Iyo, Takeru Saito, Kazuki Okada, Fumiaki Yano, Fumiaki Yamasaki, Yusuke Nakata, Tsuyoshi Sasaki, Tomihisa Niitsu

**Affiliations:** ^1^ Department of Psychiatry Chiba University Graduate School of Medicine Chiba Japan; ^2^ Department of Child Psychiatry Chiba University Hospital Chiba Japan

**Keywords:** 22q11.2 deletion syndrome, ectopic gray matter, periventricular nodular heterotopia (PNH), treatment‐resistant schizophrenia (TRS), clozapine

## Abstract

**Background:**

22q11.2 deletion syndrome is associated with schizophrenia, seizures, and often experience intolerance to antipsychotics. Periventricular nodular heterotopia (PNH) is a neuronal migration disorder that can also be observed in individuals with 22q11.2 deletion syndrome. However, to our knowledge, the use of clozapine in adolescent patients with treatment‐resistant schizophrenia and comorbid 22q11.2 deletion syndrome and PNH has not been previously reported.

**Case Presentation:**

A 17‐year‐old female with treatment‐resistant schizophrenia was referred to our hospital. She presented with auditory hallucinations, disorganized behavior, and insomnia. Multiple antipsychotics, mood stabilizers, benzodiazepines, and modified electroconvulsive therapy were either ineffective or poorly tolerated due to extrapyramidal symptoms. Brain magnetic resonance imaging (MRI) performed under sedation revealed PNH. Genetic testing confirmed a diagnosis of 22q11.2 deletion syndrome. Clozapine was initiated with close monitoring, and her symptoms gradually improved following a slow titration. She was discharged after approximately 6 months and has remained clinically stable for 15 months.

**Conclusion:**

Brain MRI and genetic testing—even when performed under sedation—may be valuable diagnostic tools in adolescents with treatment‐resistant schizophrenia. Furthermore, the presence of structural brain abnormalities does not preclude the efficacy of clozapine, which may remain a viable and effective treatment option in such cases.

## BACKGROUND

22q11.2 deletion syndrome (22q11.2 DS) is a serious genetic disorder caused by a microdeletion in the q11.2 region of Chromosome 22. It is associated with a range of congenital anomalies, including congenital heart defects, characteristic facial features, cleft palate, and juvenile‐onset Parkinson's disease.^1^ The prevalence of 22q11.2 DS is estimated to be between 1 in 2000 and 6000 live births, making it the most common chromosomal microdeletion syndrome.^2^

Neurodevelopmental and psychiatric disorders are frequently observed in individuals with 22q11.2 DS. Intellectual disability and learning difficulties are present in 70%–90% of cases. Additionally, autism spectrum disorder (ASD) and schizophrenia are reported in approximately 20% of pediatric patients and 25% of adult patients, respectively.^1^

22q11.2 DS is considered the strongest known genetic risk factor for schizophrenia, conferring more than a 20‐fold increased risk compared to the general population.^3^ Notably, nearly 60% of individuals with both schizophrenia and 22q11.2 DS exhibit treatment resistance, a significantly higher proportion than typically seen in patients with schizophrenia alone.^3^ Symptom control in this population is often challenging, as antipsychotics and mood stabilizers may be ineffective or poorly tolerated. Moreover, antipsychotic treatment can frequently induce extrapyramidal symptoms or seizures.^1^, ^4^

Gray matter heterotopia (GMH) refers to a group of neurological disorders characterized by the ectopic localization of neurons. GMH typically presents as clusters of neurons located along the ventricular walls—primarily in the form of periventricular nodular heterotopia (PNH)—or as nodules within the deep white matter (focal subcortical heterotopia), or as continuous bands of neurons within the subcortical region (subcortical band heterotopia or double cortex).^5^ Among these, PNH is considered the most severe form of GMH and is associated with a high risk of intractable epilepsy. Neuhaus et al. reported that GMH is observed in approximately 45% of patients with this syndrome, whereas PNH is identified in only 13% of cases.^6^

Here, we present the case of a patient who was initially diagnosed with schizophrenia. After being referred to our hospital, magnetic resonance imaging (MRI) revealed PNH, leading to the subsequent diagnosis of comorbid 22q11.2 DS. Following the initiation of clozapine, her psychiatric symptoms—previously unresponsive to various psychotropic medications—significantly improved. Although prior treatments failed to achieve symptom relief, clozapine resulted in marked clinical improvement.

## CASE PRESENTATION

The patient was a 17‐year‐old Japanese female. At the age of 2, developmental delays were noted during a routine infant health checkup. Subsequent intelligence testing led to a diagnosis of borderline intellectual functioning.

Upon entering elementary school, she experienced difficulties in social communication with peers and displayed signs of inattention. At the age of 14, she began expressing persecutory thoughts. She subsequently visited a psychiatric clinic. However, her symptoms had worsened; she reported being stared at and hearing others spread malicious rumors about her. Her hallucinations and delusional thinking gradually intensified.

Following auditory hallucinations instructing her to harm herself, she attempted suicide by stabbing herself in the abdomen with a kitchen knife. She was diagnosed with schizophrenia and was prescribed olanzapine 2.5 mg and risperidone 1 mg, taken separately. However, these medications had minimal effects. She was then admitted to a psychiatric hospital, but her condition showed no significant improvement. Over time, her symptoms progressively worsened, and she eventually lost the ability to communicate, even in simple interactions.

**Figure 1:**
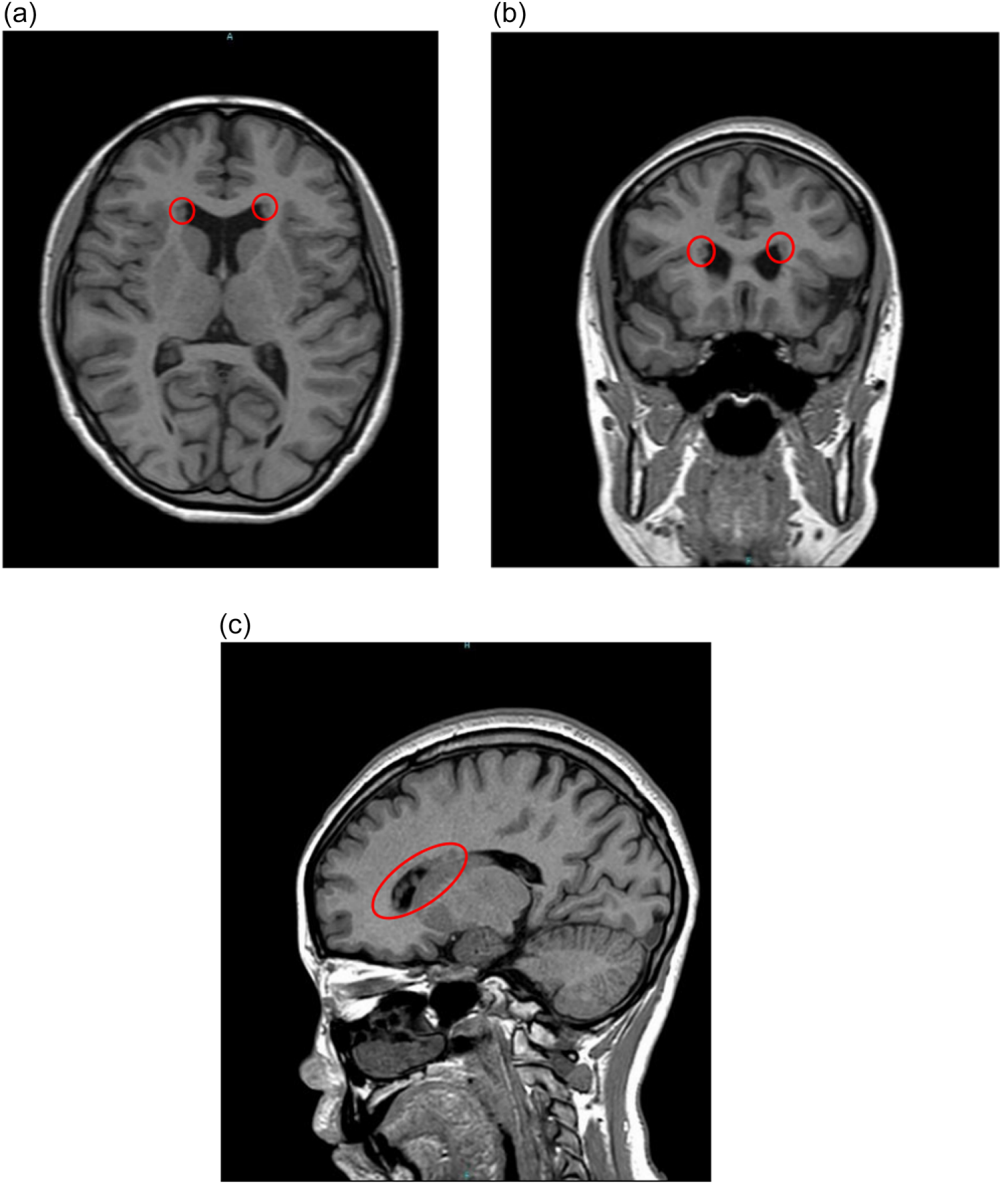
(a–c) Magnetic resonance imaging (MRI) findings. Brain MRI revealed bilateral, multiple periventricular nodular heterotopia (PNH) located at the dorsal pole of the frontal horn of the lateral ventricles. (a) Axial T1‐weighted image, (b) coronal view, and (c) sagittal view. The sagittal image (c) shows multiple PNHs along the lateral ventricular walls. The area circled in red indicates the locations of the PNHs.

At the age of 15, the patient was referred to our hospital and admitted for medical care. Due to persistent violent behavior, involuntary behavioral restrictions and isolation measures were required. A brain MRI scan was performed under sedation with flunitrazepam and revealed PNH (Figure 1). The results of genetic testing confirmed a deletion in the 22q11.2 region, consistent with 22q11.2 DS.

Despite trials with multiple antipsychotics, treatment was limited due to intolerable extrapyramidal symptoms. Other interventions, including mood stabilizers, anticonvulsants, benzodiazepines, and modified electroconvulsive therapy (mECT), were ineffective.

The patient was hospitalized multiple times, but her condition remained largely unchanged. She avoided eye contact, isolated herself in her room, repetitively sucked her fingers, and was unable to engage in even simple conversations. At times, she exhibited violent behavior toward family members, triggered by hallucinations and delusional thoughts.

In July 2023, the patient was involuntarily admitted to our hospital for medical care and protection. On Day 52 of hospitalization, clozapine was initiated for the treatment of TRS. The dose was titrated gradually and with close monitoring.

After 4 weeks of treatment, the dose was increased to 75 mg, and her impulsive and explosive behaviors began to subside. At 8 weeks, the dose was raised to 100 mg, during which time her ability to communicate improved, and she became able to engage in simple conversations. After 16 weeks, with the dose increased to 250 mg, she expressed a desire to return to school and demonstrated improved emotional expression. At 24 weeks, the dose was increased to 350 mg, and she was subsequently discharged. The blood concentration of clozapine was 503 ng/mL.

After clozapine was introduced, antiepileptic drugs were not administered. Routine electroencephalography (EEG) was performed during treatment, with no significant abnormalities detected. Her clinical progress was evaluated using the Positive and Negative Syndrome Scale (PANSS),^7^ with total scores of 160, 144, 121, 97, and 89 at Weeks 0, 4, 8, 16, and 24, respectively. Her Global Assessment of Functioning (GAF)^8^ score improved markedly, from 10 at admission to 55 at discharge, which occurred on Day 252 of hospitalization.

Following discharge, the patient was able to lead a stable daily life and resumed regular attendance at her special‐needs school. She began going out by car more frequently, eating her favorite foods at shopping malls, and communicating in simple language with visiting nurses and staff. As of 15 months post‐discharge, she has maintained remission while receiving outpatient care.

## DISCUSSION

In this case, various psychotropic medications and mECT produced minimal therapeutic benefit. In contrast, clozapine demonstrated marked efficacy. Notably, despite the presence of PNH, which is associated with a high risk of epileptic seizures, no seizures were observed during treatment.

To our knowledge, this is the first published report documenting the marked effectiveness of clozapine in the treatment of TRS in a patient with comorbid 22q11.2 DS and PNH, without any significant adverse events.

Clozapine is an atypical antipsychotic and remains the only antipsychotic medication with confirmed efficacy in TRS. Its multiple pharmacological mechanisms contribute to its superiority over other antipsychotics. Several case reports have described the successful use of clozapine in patients with schizophrenia associated with 22q11.2 DS, with a favorable response rate of approximately 65.5% across published individual cases and case series.^9^

When treating the psychiatric symptoms of 22q11.2 DS, physicians must remain vigilant about the associated risk of epilepsy.^1^ It is therefore advisable to select medications with a lower potential to induce seizures. However, Colijn reported that epilepsy is the most frequently observed serious adverse effect of clozapine in patients with 22q11.2 DS.^9^ In our case, the presence of PNH, a known risk factor for epilepsy, further increased the likelihood of seizure occurrence. As a result, we introduced clozapine with extreme caution, employing a slow titration strategy. No epileptic activity was observed either on EEG or through clinical monitoring during the post‐initiation period.

Although clozapine carries a known risk of inducing seizures, it can be highly effective in managing TRS associated with 22q11.2 DS when administered under careful observation.

**Figure 2:**
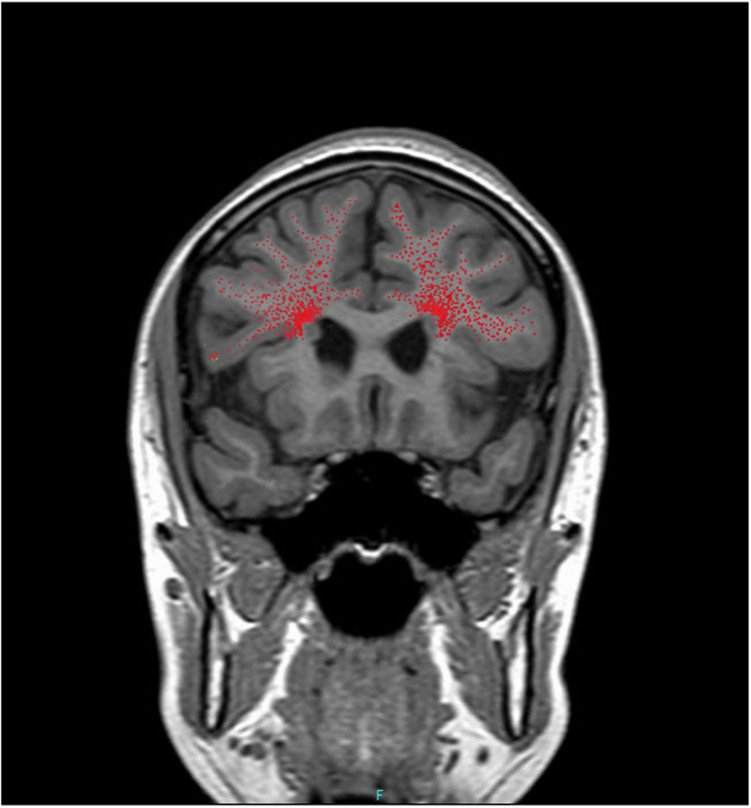
Schematic representation of bilateral arc cells migration. The red dots indicate the path of arc cells. Since arc cells extend radially toward the cortex, structural abnormalities in periventricular nodular heterotopia could be widespread.

Recent studies have identified a population of migratory neurons in the infant human brain, referred to as Arc cells. These cells originate in the subventricular zone (SVZ) and migrate around the dorsal pole of the anterior horn of the lateral ventricles (DPAHLV) toward the prefrontal cortex.^10^ This migration is organized into four anatomical tiers: Tiers 1 and 2 are located near the ventricular zone, whereas Tiers 3 and 4 extend toward the cortical surface^11^ (Figure 2). In patients with 22q11.2 DS, anatomical studies suggest that PNH may represent Arc cells that failed to migrate beyond Tiers 1 or 2. Accordingly, PNH may be regarded as one of the more severe forms of heterotopia.

Despite the presence of PNH, clozapine was effective in alleviating the patient's psychotic symptoms, suggesting its utility even in cases with severe structural abnormalities.

## CONCLUSION

We report a case of TRS comorbid with 22q11.2 DS and PNH, in which clozapine treatment was successful and no epileptic seizures were observed.

In cases of TRS, if there is no evident history of congenital anomalies such as characteristic facial features or congenital heart defects, it is advisable to conduct an organic evaluation, including brain MRI—even under sedation—and genetic testing. Importantly, the presence of structural brain abnormalities does not preclude the efficacy of clozapine, which may remain a viable and effective treatment option in such cases.

## AUTHOR CONTRIBUTIONS

Kiwamu Hoshi, Koichi Matsuyama, and Yasunori Oda wrote and reviewed the manuscript. All authors were involved in the clinical care of the patient and approved the final version of the manuscript.

## CONFLICT OF INTERESTS STATEMENT

Dr. Oda reports receiving honoraria from Otsuka, Meiji Seika, and Sumitomo Pharma. Drs. Hoshi, Iyo, Matsuyama, Saito, Okada, Sasaki, Shibata, Yano, Yamasaki, Nakata, and Niitsu report no potential conflicts of interest.

## ETHICS APPROVAL STATEMENT

This case report did not require ethics committee approval, as it did not involve research beyond standard clinical care. Written informed consent was obtained from the patient and her parents.

## PATIENT CONSENT STATEMENT

Written informed consent for presentation of the patient's clinical course was obtained from the patient and her parents.

## CLINICAL TRIAL REGISTRATION

N/A.

## Data Availability

Due to the nature of this case report, the data obtained from this report cannot be shared openly.
